# A Case of Neuroparacoccidioidomycosis in Houston, Texas

**DOI:** 10.7759/cureus.21129

**Published:** 2022-01-11

**Authors:** Maitreyi Narayan, Swetha Jayavelu, Harsh Goel, Joshua R Rosenthal, Gabriel M Aisenberg

**Affiliations:** 1 Internal Medicine, University of Texas Health Science Center at Houston, Houston, USA

**Keywords:** paracoccidioidomycosis, central nervous system infections, brain mass, paracoccidioides brasiliensis, neuroparacoccidioidomycosis

## Abstract

Paracoccidioidomycosis is a systemic fungal disease caused by the dimorphic *Paracoccidioides* species endemic to South America. Infection classically presents with pulmonary, mucosal, or reticuloendothelial involvement, though other organs can be involved.

Central nervous system involvement is rare, and almost universally reported within the endemic area for the fungus. We present a 60-year-old Brazilian male who complained of occipital headache, ataxia, dysmetria, and dysarthria for two months, diagnosed with neuroparacoccidioidomycosis in Houston, Texas. The patient had a cerebellar mass and a left pulmonary spiculated apical mass suspicious for a lung metastatic malignancy and a preliminary histological report consistent with invasive cryptococcosis. The patient’s work and travel history were paramount in achieving the final diagnosis.

## Introduction

Paracoccidioidomycosis is a systemic fungal disease caused by the dimorphic *Paracoccidioides *species [1-3]. This fungal species is mainly found in the soil of South America, with 80% of cases reported from Brazil, especially in those who are continually exposed to agriculture such as rural workers [1]. Neuroparacoccidioidomycosis, the central nervous system (CNS) compromised by this fungus, has only been reported in endemic areas except for a delayed relapse in a person who moved from Brazil to the United States [4]. Here, we present a male patient admitted in Houston, Texas with occipital headache, ataxia, dysmetria, and dysarthria, diagnosed with neuroparacoccidioidomycosis. The patient’s social and occupational histories were the main diagnostic clues in a case confounded by concerns for malignancy and a cryptococcal infection.

## Case presentation

A 60-year-old male presented to the hospital with severe occipital headache, ataxia, and dysarthria, progressive for two months. He had been a rice farmer in Sao Paulo, Brazil, and arrived in the United States two days prior to visit his daughter. He has a 50-pack a year smoking history. The patient was afebrile with a blood pressure of 110/77 mmHg, heart rate of 46 beats per minute, respiratory rate of 13 breaths per minute, and 96% of oxygen saturation on ambient air. Physical examination was significant for slurred speech, truncal ataxia, bilateral dysmetria, and dysdiadochokinesia. The remainder of the examination (cardiac, respiratory, and abdominal) was normal. Basic laboratory tests were remarkable for mildly elevated transaminases with an unremarkable white count (Table 1).

**Table 1:** Pertinent laboratory results. g/dL = grams per deciliter; k/μL = thousand per microliter; % = percent; AST = aspartate transaminase; ALT = alanine transaminase; L = liter.

Laboratory	Result	Reference
Hemoglobin	14.4 g/dL	14-18 g/dL
White count	10.5 k/μL	3.7-10.4 k/μL
Neutrophils	78.4%	45%-75%
Lymphocytes	12.2%	20%-40%
Monocytes	7%	2%-12%
Basophils	1.4%	0%-1%
Eosinophils	1%	0%-4%
AST	139 unit/L	0-65 unit/L
ALT	54 unit/L	0-37 unit/L
HIV	Negative	Negative

**Figure 1:**
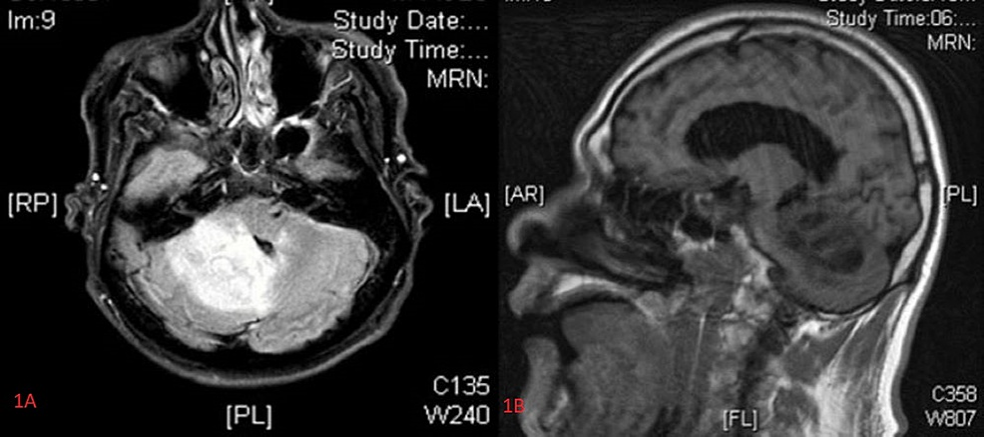
Magnetic resonance images of the cerebellum (T1 mode). Axial and sagittal images showing a cerebellar mass.

**Figure 2:**
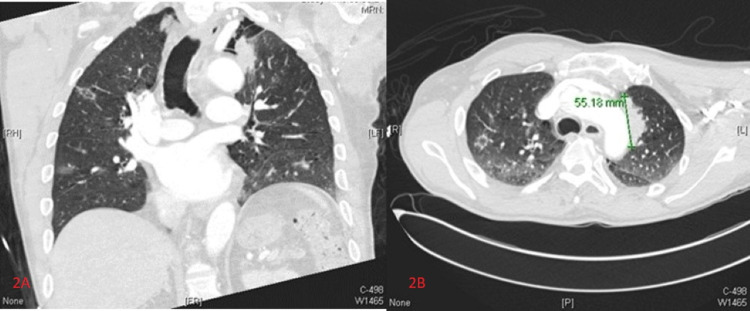
Chest computed tomography with iodine contrast. Coronal and axial images showing a spiculated mass in the left apical lung prior to antifungal therapy.

Human immunodeficiency virus (HIV) serology was negative. Brain magnetic resonance imaging (MRI) with and without contrast revealed multiple enhancing cerebellar lesions (Figures 1A, 1B). A computed tomography (CT) of the chest with contrast revealed an irregular, spiculated left apical lung mass measuring 5.5 x 3.7 x 4.1 cm (Figures 2A, 2B), with multiple smaller bilateral spiculated lung nodules, ground-glass opacities, and right hilar lymphadenopathy concerning for metastatic malignancy.

The neurosurgery team completed a large open hemicraniectomy with ultrasound navigation. During resection, they noted many deposits along the cerebellar dura and a significant amount of leptomeningeal disease along the superior surface of the right cerebellum. A preliminary pathology report showed evidence of pseudo-cystic fungal disease morphologically consistent with *Cryptococcus neoformans* and without evidence of malignancy. Empiric treatment with amphotericin B and flucytosine was started.

A lumbar puncture was performed, which showed an unremarkable opening pressure, lymphocytic pleocytosis, elevated protein, low normal glucose, and negative cryptococcal antigen (Table 2).

**Table 2:** Lumbar puncture studies. CSF = cerebrospinal fluid; cm = centimeter; H_2_0 = water; mg/dL = milligram per deciliter; mm^3^ = cubic millimeter; % = percent.

Laboratory	Result	Reference
CSF opening pressure	10 cm H_2_0	7 -18 cm H_2_0
CSF glucose	40 mg/dL	45-80 mg/dL
CSF total protein	172 mg/dL	15-45 mg/dL
CSF white count	20 white cells/mm3	0-5 white cells/mm^3^
CSF lymphocytes	92%	40%-80%
CSF neutrophils	4%	0%-6%
CSF monocytes	4%	15%-45%
Cryptococcal antigen	Negative	Negative

**Figure 3:**
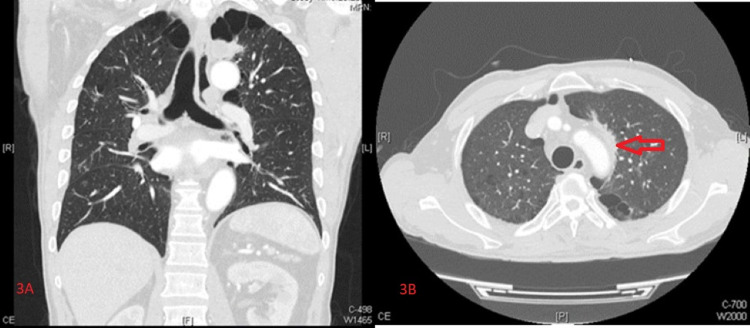
Chest computed tomography with iodine contrast. Coronal and axial images showing a reduction in the size of the left apical mass after antifungal therapy.

Cultures were negative for bacteria and fungi. Given the negative serology for HIV, negative cryptococcal antigen*,* and an exposure history concerning for *Paracoccidioides brasiliensis*, cultures for the latter were sent to a specialized laboratory. On day 18 of the course of amphotericin B, final fungal cultures revealed an infection of *Paracoccidioides brasiliensis,* and treatment was switched to oral voriconazole 200 mg twice daily. A repeat CT of the chest obtained after 18 days of antifungal therapy and 25 days after prior chest imaging showed a significant reduction in the size of the spiculated apical lung mass (Figures 3A, 3B).

The patient was discharged on voriconazole with instructions for outpatient follow-up with infectious disease and pulmonology for assessment of further response to antifungal therapy with possible biopsy of the left apical lung mass.

## Discussion

This case describes a rare manifestation of paracoccidioidomycosis outside of its endemic area with misleading radiological and pathological characteristics.

Classically, paracoccidioidomycosis has two major manifestations: an acute or subacute form and a chronic form [5]. The acute or subacute form, known as the juvenile type, occurs in young adults and children [2,5] and presents typically with lymphadenopathy, ulcerations of the skin and mucosal membranes, hepatomegaly, and splenomegaly. The chronic form, or adult form, is more common and occurs after the age of 30 years. The manifestations occur years after initial exposure to the fungus [2]. This form presents with oropharyngeal lesions and predominant pulmonary involvement [5]. The chronic form, more common in smokers and those who consume alcohol beyond 50 g/day, is typically observed in endemic areas among agricultural workers [5,6].

Neuroparacoccidioidomycosis, however, is quite rare, noted in about 10-27% of all cases [7-9]. It presents in one of three forms: a meningeal form, a combined meningeal pseudo-tumoral form, and a parenchymal pseudo-tumoral form, the latter of which is more common [10]. Isolated meningitis is rare, but it can be associated with a pseudo-tumoral presentation [11].

Neurological involvement of this disease is often secondary to hematological dissemination from lesions in areas with high blood flow. Of these primary lesions, 61% to 83% of patients present with pulmonary lesions, and 5% and 3.1% of patients with osteoarticular involvement and involvement of the digestive tract, respectively [12]. A systematic review noted that within the CNS, the most affected regions include the cerebral hemispheres (47.6%), with primarily frontal and parietal lobe involvement, and the cerebellum (28.8%) [10,12].

In our case, due to the patient’s long-standing smoking history and the findings of a spiculated lung mass with multiple cerebellar masses, primary lung malignancy with CNS metastasis was suspected as opposed to primary paracoccidioidomycosis with CNS involvement. Epidemiologically, lung cancer is far more common than paracoccidioidomycosis in the United States, with more than 1.8 million new cases projected for 2021 [13]. Comparatively, paracoccidioidomycosis only has a couple of dozen reported cases in the United States [1]. Given the significant reduction in the size of the spiculated mass following initial treatment with amphotericin B, our patient’s presentation was most likely attributable to pulmonary paracoccidioidomycosis rather than malignancy. This ultimately reflects the high degree of clinical suspicion required to diagnose paracoccidioidomycosis in non-endemic areas, as well as the challenges with its diagnosis in the presence of clinical confounders (extensive smoking history and spiculated lung mass).

Commencing appropriate and early targeted therapy for paracoccidioidomycosis in our case was also challenged by an initial suspicion for *Cryptococcus* and the lack of readily available targeted diagnostics. In our case, given the patient’s social history, negative HIV serology, and negative cryptococcal studies, a suspicion for paracoccidioidomycosis was raised despite pathological concern for *Cryptococcus neoformans*. However, while treatment of cryptococcal infections requires intravenous amphotericin and flucytosine, followed by fluconazole, treatment options for paracoccidioidomycosis are much broader, including azole derivatives, amphotericin B, terbinafine, and even trimethoprim-sulfamethoxazole (TMP-SMX) [14-16].

Morphologically, *Cryptococcus* is a dimorphic fungus that grows in a single-budding yeast form with a distinct polysaccharide capsule [17]. In contrast, *Paracoccidioides* takes a spherical, multiple-budding yeast form that resembles that of a ship’s wheel, earning the term “Captain’s Wheel.” However, non-budding or single-budding forms can dominate, which can lead to misdiagnosis [18,19]. Stained with Gomori’s methenamine silver, these atypical forms have been confused with other yeast-like pathogens such as *Candida*, *Cryptococcus*, or *Pneumocystis* [18-20]. In patients presenting with symptoms concerning an endemic fungal infection and an absence of characteristic pathological morphology, serologic testing can be performed using immunodiffusion and complement-fixing methods to detect antibodies against the fungal pathogen in question [18].

## Conclusions

This case report explores a rare neurologic manifestation of paracoccidioidomycosis at a hospital in Houston, Texas. Paracoccidioidomycosis is an important consideration in the diagnosis of infectious etiologies, even in immunocompetent patients, and especially in patients from endemic South America. The diagnosis can be challenging in non-endemic areas due to unfamiliarity with the pathogen, limited availability of diagnostic tests, and the presence of clinical confounders, which may skew the differential diagnosis. A thorough social history, including travel and occupational history, can assist in making the correct diagnosis.
